# Patellar Tendon Rupture Following Total Knee Arthroplasty: A Case Report

**DOI:** 10.7759/cureus.100526

**Published:** 2025-12-31

**Authors:** Arif Akkok, John Sawires, Asli Akkok, Anthony Horvath

**Affiliations:** 1 Orthopaedic Surgery, Lake Erie College of Osteopathic Medicine, Elmira, USA; 2 Anaesthesiology and Critical Care, Lake Erie College of Osteopathic Medicine, Elmira, USA; 3 General Surgery, CUNY School of Medicine, New York, USA; 4 Orthopaedic Surgery, St. John's Episcopal Hospital, New York, USA

**Keywords:** achilles tendon allograft, extensor mechanism disruption, patellar tendon rupture, reconstruction, total knee arthroplasty

## Abstract

Patellar tendon rupture following total knee arthroplasty (TKA) is a rare but serious complication that has the ability to result in significant functional impairment due to extensor mechanism disruption. In the early postoperative period, diagnosis may be challenging, especially if initial radiographs are unremarkable and some function is preserved. We present the case of a 60-year-old female who presented to the emergency department with right-sided knee pain following a mechanical fall approximately one month after undergoing right TKA. Initial evaluation showed intact prosthetic components, the ability to bear weight with assistance, and preserved passive range of motion, leading to discharge. Persistent pain prompted further outpatient evaluation. Magnetic resonance imaging subsequently demonstrated a complete rupture of the patellar tendon. The patient underwent surgical reconstruction with an Achilles tendon allograft and tolerated the procedure without any complications. This case highlights the potential for delayed diagnosis of patellar tendon rupture in a patient who recently underwent TKA. It also supports Achilles tendon allograft reconstruction as a viable and reliable option when primary repair is not feasible.

## Introduction

Total knee arthroplasty (TKA) is a common and successful procedure performed for the management of advanced degenerative knee disease. Although most patients experience significant functional improvement and pain relief following surgery, rare but potentially devastating complications involving the extensor mechanism can occur. Extensor mechanism disruptions following TKA, such as patellar fracture, quadriceps tendon rupture, and patellar tendon rupture, occur in less than 1% of cases. Patellar tendon rupture is the least common yet most clinically challenging of these complications [1,2].

Patellar tendon rupture following TKA leads to significant functional disability, including gait instability, loss of active knee extension, and reduced overall knee function. Major risk factors are traumatic injury, recent arthroplasty, infection, and impaired tendon integrity. Early radiographs can show intact prosthetic components, and it is possible for patients to regain partial extensor function, masking the severity of injury and delaying diagnosis [3,4].

Patellar tendon rupture management following TKA remains a clinical challenge. Reconstruction using autograft/allograft tissue has become the mainstay treatment strategy in many cases, as primary repair alone has been associated with high failure rates due to increased stress across the repair site and poor tissue quality in the post-surgical knee [5]. Achilles tendon allograft reconstruction is a reliable option, providing strong tendon length and a calcaneal block that permits secure tibial fixation while also facilitating restoration of patellar height and maintaining extensor mechanism continuity [6-7].

## Case presentation

A 60-year-old female presented to the emergency department with right knee pain following a mechanical fall. She noted immediate pain but denied numbness and was able to ambulate with assistance. Her past surgical history was significant for a right TKA performed one month prior. She had an unremarkable postoperative course. On physical examination, she demonstrated a full passive range of motion of the right knee and was able to bear weight with assistance. Mild swelling was noted without erythema, warmth, or signs of infection. Radiographs showed intact prosthetic components without evidence of fracture, loosening, or malalignment. As a result, she was discharged with conservative management. 

At a subsequent outpatient orthopedic clinic visit, she reported persistent anterior knee pain and subjective weakness with ambulation. Physical examination revealed extensor mechanism insufficiency, prompting further evaluation. Advanced imaging supported the diagnosis of patellar tendon rupture, which was definitively confirmed during surgical exploration. She was counseled on treatment options, specifically reconstruction using an Achilles tendon allograft, and chose to proceed with surgery.

A standard anteromedial approach was used, exposing extensive scar tissue and the deficient patellar tendon remnant. A lateral retinacular release was performed to mobilize the extensor mechanism, improving patellar mobility and alignment. A tibial trough measuring 2.5-3 cm × 2 cm was fashioned at the level of the tibial tuberosity. The cortical-cancellous bone block of the Achilles tendon allograft was seated into the trough and secured with a 4 mm partially threaded cancellous screw to ensure rigid fixation. 

The tendon was split into three longitudinal branches. The central portion was anchored to the distal pole of the patella with two suture anchors and further woven into the quadriceps tendon at the superior patellar pole. The medial and lateral branches were tacked to their respective retinacula to restore extensor mechanism balance and reinforce the reconstruction. The native patellar tendon stump was incorporated into the graft construct in order to promote biologic integration. Intraoperative assessment demonstrated appropriate patellar height and stability of the reconstruction. Knee flexion to 90 degrees was achieved without excessive tension. Standard layered closure was performed. 

**Figure 1:**
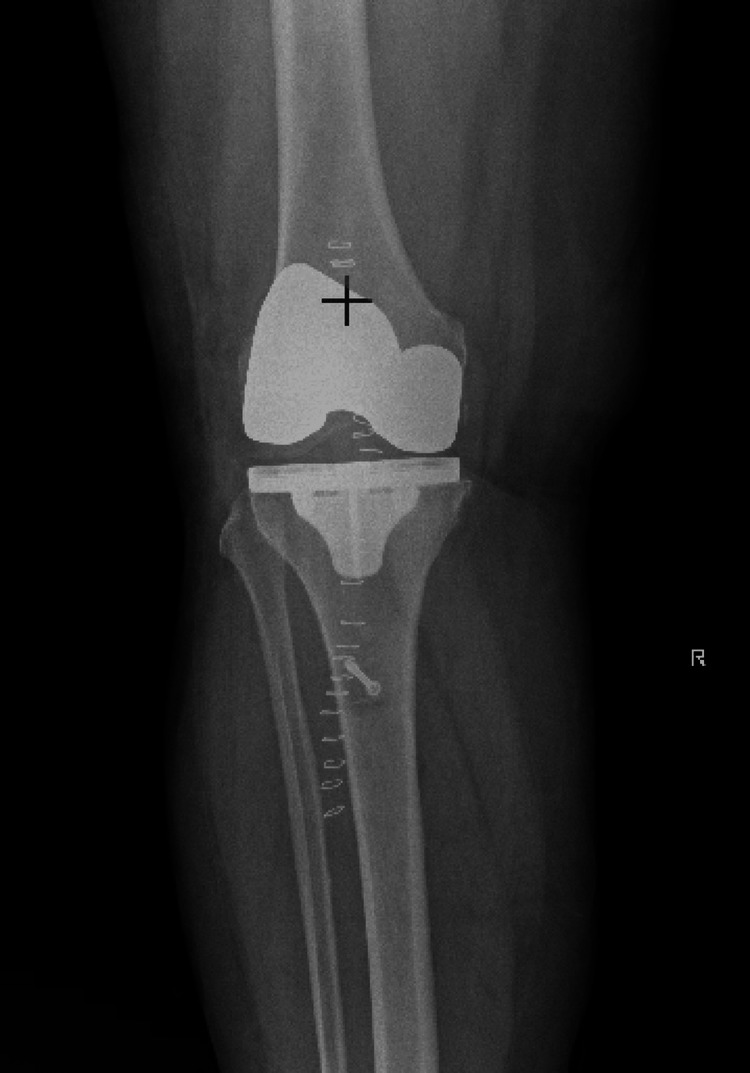
Postoperative anteroposterior radiograph of the right knee Postoperative anteroposterior radiograph of the right knee demonstrating intact total knee arthroplasty components with restored patellar height following Achilles tendon allograft reconstruction of the patellar tendon.

**Figure 2:**
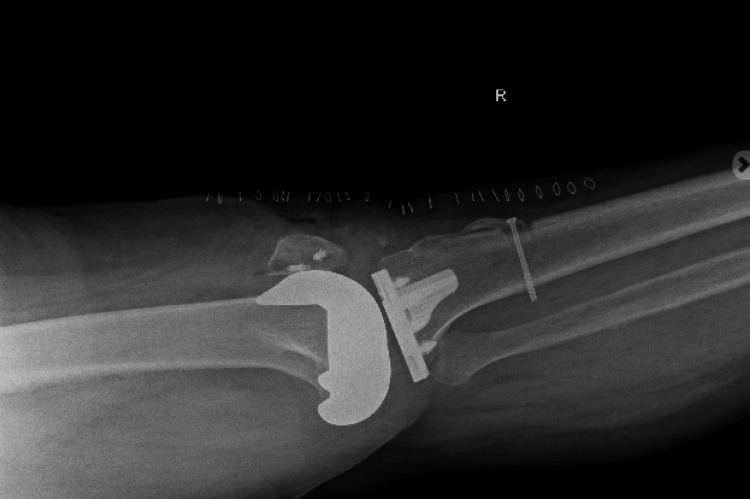
Postoperative lateral radiograph of the right knee Postoperative lateral radiograph of the right knee demonstrating intact total knee arthroplasty components with restored patellar height following Achilles tendon allograft reconstruction of the patellar tendon.

The procedure was tolerated well without any intraoperative or immediate postoperative complications. Extensor mechanism continuity and patellar height were confirmed intraoperatively. Postoperative radiographs demonstrated intact TKA components with restoration of patellar height following Achilles tendon allograft reconstruction (Figures 1-2). Postoperatively, the patient was maintained in a knee immobilizer locked in extension and remained strictly non-weight bearing on the operative extremity, using a wheelchair for mobility during early follow-up. At her postoperative clinic visit, she demonstrated improved knee stability and restoration of active extension. There were no signs of infection, graft failure, or wound complications. At her postoperative clinic visit, she demonstrated improved knee stability and restoration of active extension. There were no signs of infection, graft failure, or wound complications.

## Discussion

Patellar tendon rupture following TKA is a rare but devastating complication due to its vital role in maintaining extensor mechanism integrity. It represents a technically demanding problem without marked consensus regarding optimal management. In the present case presentation, diagnosis was delayed due to the ability to bear weight with assistance, preserved passive motion, and initially reassuring radiographs. This case highlights the limitations of standard imaging modalities in discovering early extensor mechanism disruption. In addition, it underscores the significance of maintaining a high index of suspicion in patients presenting with constant anterior knee weakness or pain after recent TKA, especially following trauma. 

Once diagnosis is established, viable treatment options must consider the altered biological environment and compromised biomechanics of the post-arthroplasty knee. Primary repair has been associated with high failure rates, mostly due to increased tensile forces across the extensor mechanism and poor tissue quality [8-9]. Therefore, reconstruction with allograft tissue has become the favored strategy in many reported cases, specifically in cases of delayed diagnosis or inadequate native tendon. In the present case, amalgamation of the graft into the native patellar tendon remnant and quadriceps tendon, coupled with reinforcement of the medial and lateral retinacula, was done to enhance construct stability and load sharing. Restoration of extensor continuity and appropriate patellar tracking through knee flexion was confirmed intraoperatively. Reconstruction with the use of an Achilles tendon allograft offers several advantages in this context. The calcaneal bone block permits for rigid fixation within the tibia. The length and strength of the tendon allow restoration of patellar height and calibrated tensioning of the extensor mechanism [10,11].

Surgical technique and execution play a significant role in the success of extensor mechanism reconstruction. Precise positioning of the tibial trough, firm fixation of the bone block, and meticulous attention to graft tension are essential to avoid complications like patella baja, extensor lag, or even graft failure [10-12]. Despite the advances in reconstructive techniques, extensor mechanism reconstruction following TKA is still associated with higher complication rates relative to primary arthroplasty procedures. Therefore, individualized surgical planning, careful postoperative rehabilitation, and patient counseling are imperative. This case emphasizes that Achilles tendon allograft reconstruction can effectively restore extensor mechanism continuity in a delayed diagnosis, given that meticulous surgical technique is utilized.

## Conclusions

Patellar tendon rupture following TKA may present subtly and can be potentially overlooked in the early postoperative period due to normal radiographs and preserved passive motion. This present case highlights the importance of heightened clinical vigilance and advanced imaging in a timely manner when extensor mechanism injury is suspected after TKA. Achilles tendon allograft reconstruction offers an effective solution for restoring extensor mechanism continuity, especially when primary repair is not feasible. Vigilance of this complication and prompt, definitive management are essential to prevent long-term functional impairment.
